# Prospective development study of the Versius Surgical System for use in transoral robotic surgery: an IDEAL stage 1/2a first in human and initial case series experience

**DOI:** 10.1007/s00405-024-08564-6

**Published:** 2024-03-26

**Authors:** Jack Faulkner, Asit Arora, Peter McCulloch, Stephen Robertson, Aleix Rovira, Sebastien Ourselin, Jean-Pierre Jeannon

**Affiliations:** 1 School of Biomedical Engineering and Imaging Sciences, King’s College London, London, UK; 2 Department of Head and Neck Surgery, Guy’s and St Thomas’ NHS Foundation Trust, London, UK; 3 Nuffield Department of Surgical Science, University of Oxford, Oxford, UK

**Keywords:** TORS, Transoral robotic surgery, Robotic, Minimally invasive surgery, Head and neck

## Abstract

**Purpose:**

Transoral robotic surgery is well established in the treatment paradigm of oropharyngeal pathology. The Versius Surgical System (CMR Surgical) is a robotic platform in clinical use in multiple specialities but is currently untested in the head and neck. This study utilises the IDEAL framework of surgical innovation to prospectively evaluate and report a first in human clinical experience and single centre case series of transoral robotic surgery (TORS) with Versius.

**Methods:**

Following IDEAL framework stages 1 and 2a, the study evaluated Versius to perform first in human TORS before transitioning from benign to malignant cases. Iterative adjustments were made to system setup, instrumentation, and technique, recorded in accordance with IDEAL recommendations. Evaluation criteria included successful procedure completion, setup time, operative time, complications, and subjective impressions. Further evaluation of the system to perform four-arm surgery was conducted.

**Results:**

30 TORS procedures were successfully completed (15 benign, 15 malignant) without intraoperative complication or conversion to open surgery. Setup time significantly decreased over the study period. Instrumentation challenges were identified, urging the need for TORS-specific instruments. The study introduced four-arm surgery, showcasing Versius’ unique capabilities, although limitations in distal access were observed.

**Conclusions:**

TORS is feasible with the Versius Surgical System. The development of TORS-specific instruments would benefit performance and wider adoption of the system. 4-arm surgery is possible however further evaluation is required. Multicentre evaluation (IDEAL stage 2b) is recommended.

**Supplementary Information:**

The online version contains supplementary material available at 10.1007/s00405-024-08564-6.

## Introduction

Since its introduction in 2005 transoral robotic surgery has become well established in the treatment paradigm of benign and malignant head and neck pathology. TORS offers a minimally invasive surgical option and applies the latest advances in technology to enhance the surgical experience. Three-dimensional high definition optics, tremor filtration, motion scaling and enhanced instrument range of motion allow en-bloc transoral tumour resections to be performed without the morbidity of open techniques [1–3].

Adoption of transoral robotic surgery is rapidly expanding, however, robotic head and neck service development can be limited by cost effectiveness and robotic platform availability and access issues. Multiple new robotic platforms are entering the market and are becoming commercially available. This increase in competition may help to further drive down costs and increase adoption of robotic technology [4].

The Versius Surgical System (CMR Surgical Ltd., Cambridge, UK) is a clinically available modular robotic system that is currently approved for use in general surgery, gynaecology, thoracic and urological procedures [5–12]. Versius uses a novel modular design with multi-jointed instrument arms and a visualisation arm mounted on individual bedside units (BSU). The system can be setup in multiple configurations tailored to the required task. Each BSU is individually portable and can be transported between theatres and hospital sites. The operating surgeon interacts with the system through an open console which utilises polarised glasses to provide three-dimensional high definition optics. The system is operated through hand controls and the console is adjustable allowing the console surgeon to sit or stand.

Versius is not currently CE marked or FDA approved for use within the head and neck and has not been clinically tested in this environment. Our study team has conducted pre-clinical dry lab and cadaveric evaluation of the system for transoral use completing a stage 0 assessment in accordance with the IDEAL framework of surgical innovation and concluded that clinical assessment of the system is appropriate however further development of instrumentation would be desired for wider clinical dissemination [7, 13].

The IDEAL collaborative recognises the challenges of evaluating new surgical techniques and devices and has developed and established a framework by which surgical innovation should be performed (Table 1). IDEAL recommend the use of a Prospective Development Study for reporting early stage studies in order to provide clarity of process and clear reporting when techniques are undergoing rapid iterative changes [5–7]. Prospective Development Studies report outcomes sequentially and are transparent about any modifications to techniques throughout the study. This study presents a first in human and small cohort prospective development study of the use of the Versius Surgical System to perform transoral robotic surgery in accordance with the IDEAL framework stages 1 and 2a.

**Table 1:** The IDEAL framework stages of innovation [6, 7]

Stage 0 Pre-IDEAL	Stage 1 Idea	Stage 2a Development	Stage 2b Exploration	Stage 3 Assessment	Stage 4 Long term monitoring
Pre-clinical	First in Human	Single Centre case series/prospective cohort	Bridge from observational to comparative evaluation. Purpose is to gain data to decide if and how to test in a robust RCT or other appropriate pivotal design	Definitive comparative evaluation of main efficacy and safety aspects of new technique against current best treatment	
Feasibility and definition of procedure Simulation, Cadaveric, Animal, Modelling, Cost studies	Initial report Innovation may be planned, accidental or forced Focus on explanation and description	“Tinkering” (Rapid iterative modification of technique and indications) Small experience from one centre Focus on technical details and feasibility	Technique now more stable Replication by others Focus on adverse effects and potential benefits Learning curves important Definition and quality parameters developed	Gaining wide acceptance Considered as possible replacement for current treatment Comparison against current best practice	Monitoring late and rare problems, changes in use

## Methods

### Study protocol

A prospective single centre cohort study was performed at a UK tertiary head and neck centre to evaluate the clinical use of the Versius Surgical System (CMR Surgical) to perform transoral robotic surgery. The study encompassed a world first in-human TORS procedure with Versius (IDEAL Stage 1) and an initial case series (IDEAL Stage 2a) during which iterative changes were made to evaluate and optimise the system for use in a spectrum of transoral procedures.

In order to reduce surgical risks, the study was designed to begin with five benign procedures followed by formal review and, if appropriate, proceed to malignant surgical procedures. All cases were selected by the operating surgeon and eligibility criteria were met if they were deemed suitable for transoral robotic surgery, including adequate mouth opening and for malignant cases T1 or T2 oropharyngeal disease. All patients were consenting adult patients. Exclusion criteria were vulnerable patients including prisoners, those with severe concomitant comorbidities that either reduced life expectancy or increased risk of therapeutic interventions and patients not suitable for transoral surgery due to poor mouth opening or other anatomical limitation. No additional restrictions were present.

This study followed the principle of prospective development whereby iterative adjustments and changes to the surgical procedure occurred including changes to system setup and operative technique. This study followed IDEAL Recommendations and all changes or adjustments to the system or procedure were recorded. Following each case a formal debrief occurred during which the procedure and amendments were discussed and suitable changes carried forward into subsequent cases. Subjective impressions were also recorded and utilised to amend practice.

### Preparation and prior experience

All procedures were performed at Guy’s Hospital, London. The head and neck surgical team at Guy’s Hospital London established its transoral robotic surgery programme in 2018. The department has three fully trained TORS surgeons including one of two UK-based TORS proctors (AA). The unit performs approximately 100 transoral robotic cases annually including benign, malignant and salvage procedures and has clinical experience utilising several iterations of the Da Vinci family of robotic platforms. The surgical team has experience with the Versius Surgical System (CMR Surgical) in the pre-clinical setting. The authors (AA, JPJ, JF) undertook pre-clinical (IDEAL-D stage 0) evaluation of Versius over a 3-year period between 2019 and 2022. This pre-clinical assessment involved TORS-specific dry lab and cadaver-based evaluation and optimisation of the system as well as assessment by a total of 11 experienced TORS surgical consultants prior to proceeding to first in human clinical assessment.

In addition to undertaking pre-clinical cadaveric assessments the surgical team, including theatre staff and surgeons, completed system specific online training, hands on system training and assessments as well as a full simulated first procedure and simulated emergencies in theatre prior to the first clinical case. Surgeons additionally undertook a dedicated robotic simulation training programme with performance-based metrics and were required to meet the minimum competency level prior to proceeding with clinical cases. All system and simulation-based training was approved and overseen by CMR Surgical. Representatives from CMR Surgical were present in theatre for all cases and could assist with any system related issues.

### Surgical technique

Preclinical evaluation established feasibility for three key index TORS procedures (lateral oropharyngectomy, tongue base resection and partial supraglottic laryngectomy). This study evaluates the system to clinically perform surgery in benign oropharyngeal pathology suitable for transoral resection. Following successful evaluation of surgery for benign disease, the study evaluated the use of Versius in a variety of cancer cases including carcinoma of unknown primary tonsillectomy and tongue base mucosectomy, transoral biopsy, lateral oropharyngectomy, and revision cancer cases.

All patients were prepared and positioned as per the departments standard practice for TORS patients. This included all patients undergoing nasal intubation to ensure maximal transoral access and positioning on the operating table with a shoulder roll and head ring to facilitate access.

All cases were performed utilising a Boyle-Davis oral retractor with an unsplit appropriately sized Doughty blade. A hard plastic cheek and lip retractor protected the patients’ lips.

Once positioned the bedside units were placed around the bedside based on the optimised setup defined through preclinical evaluation (image 1) [13]. The surgeon console is positioned so that the operating surgeon is easily able to see the robotic arms and communicate directly with the bedside surgeon and theatre team.

### Virtual pivot point

Once the bedside units are positioned each unit must be orientated and a virtual pivot point (VPP) defined. The VPP concept is unique to the Versius system as the instruments in TORS operate without the use of instrument trocars and do not have a physical point around which the instruments can rotate. Preclinical evaluation determined the optimal VPP position for TORS to have the endoscope placed centrally at the level of the incisors and the instruments laterally at the midpoint of the lip and cheek retractor (Fig. 2).

### Instrumentation

The Versius surgical system utilises a 12mm endoscope available with a 0 or 30 degree viewing angle and 6mm instruments. 0 and 30 degree endoscopes are used as standard in TORS practice and the same viewing angle endoscope was selected for each procedure as if it was performed with an alternative robotic system. The available 6mm instruments differs from those available and typically utilised in TORS with the Da Vinci family of robots. Pre-clinical evaluation determined that the currently available instrument set was feasible but suboptimal for transoral surgery. The Versius instrument set does not currently feature a monopolar spatula which is the primary dissecting instrument in clinical use in TORS with alternative systems. Preclinical evaluation determined that TORS was feasible with the current instrument setup, however, a monopolar spatula would be highly desirable for wider dissemination of the system. This study evaluated the clinical applicability of the current instruments available with Versius (Table 2).

**Table 2:** Versius surgical system instruments

Instrument	Monopolar electrocautery	Bipolar electrocautery
Fenestrated grasper	No	No
Maryland grasper	No	Yes
Dissecting hook	Yes	No
Curved Scissors	Yes	No

### Outcome measures

The primary outcome measure was successful completion of each surgical procedure in the absence of significant intraoperative adverse event and absence of post-operative complication with a Clavien–Dindo score greater than 2.

Secondary outcome measures were setup time, operative time, surgical margins, intraoperative and 30-day post-operative complications and function outcome assessment via MD Anderson Dysphagia Index were all recorded. Any changes to theatre setup or surgical technique was noted. Following each procedure a formal debrief was undertaken and appropriate iterative changes noted prior to the next procedure.

## Results

30 patients underwent transoral robotic procedures with the Versius Surgical System in an 8-month period from December 2022 to August 2023. The cohort consisted of 11 women and 19 men with an age range of 19–75 year (median 51 years). 17 patients had benign pathology and 13 malignant. 28 procedures were performed by a single consultant surgeon and 2 cases performed by a trainee under supervision. There were no conversions to open surgery and no intraoperative complications were recorded and estimated blood loss was minimal for all procedures. Post-operatively one patient had persistent tongue numbness and taste disturbance at 30 days (Clavien–Dindo grade 1) and 2 benign tonsillectomy patients experienced secondary haemorrhages, both managed conservatively (Clavien–Dindo grade 2). Table 3 gives details of sequential patients, operations performed, instruments trialed, setup time, operative time and complications.

**Table 3:** Consecutive case summary of Transoral robotic surgery procedures performed with Versius Surgical System

Case number	Procedure	Benign/Malignant	Setup time (minutes)	Operative time (minutes)	Instruments trialled	Complication	Clavien Dindo classification of complication	Surgeon notes/comments
1	Bilateral tonsillectomy	Benign	24	40	Fenestrated graspers, Monopolar scissors	No		Electrocautery from monopolar scissors not effective Fenestrated graspers too large for use
2	Vallecular cyst excision	Benign	18	41	Monopolar scissors, BMG	No		Monopolar scissors insulating sheath obscured visibility of instrument tip in tongue base
3	Tongue base mucosectomy	Benign	18	107	Monopolar scissors, BMG	Postoperative tongue numbness and dysgeusia. Still present at 30 days	CD1	Electrocautery not reliable on Bipolar Maryland Grasper
4	Unilateral tonsil lesion excision	Benign	17	47	BMG, monopolar Hook	No		Bipolar Maryland Grasper not delivering electrocautery
5	Bilateral tonsillectomy	Benign	13	70	BMG, Monopolar scissors, Monopolar Hook	No		Intermittent electrocautery delivery with monopolar scissors – changed to hook
6	Bilateral tonsillectomy	Benign	5	48	Fenestrated graspers, monopolar Hook	No		Fenestrated graspers cumbersome within oropharynx. No bipolar available. Haemostasis ineffective with monopolar alone. Bedside surgeon used hand held bipolar
7	Oropharyngeal excision biopsy	Benign	20	17	BMG, Monopolar hook	No		BMG and hook worked well in combination. No issues with electrocautery
8	Lateral oropharyngectomy	Malignant	10	117	BMG, Monopolar hook	No		Setup and equipment performed effectively
9	Unilateral tonsil biopsy	Malignant	10	51	BMG, Monopolar Scissors	No		BMG ineffective at grasping tumour – tips misaligned. Monopolar scissors too bulky
10	Bilateral tonsillectomy	Benign	6	45	Right tonsil—BMG, Monopolar scissors Left tonsil—BMG, Monopolar hook	No		Hook allowed more precise electrocautery delivery than Monopolar scissors
11	Revision oropharyngectomy	Malignant	6	102	BMG, Monopolar Hook	No		No issues encountered
12	Lateral oropharyngectomy	Malignant	6	106	BMG, Monopolar Hook	No		Range of motion of instruments favourable. BMG difficult to apply effective bipolar energy due to tip control
13	Unilateral tonsillectomy	Benign	14	40	BMG, Monopolar Hook	Secondary haemorrhage day 5 post procedure. Managed conservatively	CD2	No issues encountered
14	Bilateral tonsillectomy	Benign	13	17	BMG, Monopolar Hook	No		No issues encountered
15	Bilateral tonsillectomy	Benign	12	60	BMG, Monopolar Hook	No		No issues encountered
16	Unilateral tonsil biopsy	Malignant	12	33	BMG, Monopolar Hook	No		No issues encountered
17	Tongue base mucosectomy	Malignant	10	147	BMG, Monopolar Hook	No		Good access to tongue base. Ability to control jaw opening of BMG difficult. Monopolar hook difficult to use in tongue base
18	Unilateral tonsillectomy	Malignant	6	158	BMG, Monopolar Hook	No		No issues encountered
19	Tongue base mucosectomy	Malignant	13	92	BMG, Monopolar Hook	No		Difficult to access tongue base with monopolar hook
20	Lateral oropharyngectomy	Malignant	9	108	BMG, Monopolar Hook	No		No issues encountered
21	Oropharyngeal excision biopsy	Benign	12	14	BMG, Monopolar Hook	No		Trial of 4 surgical arms—good access
22	Unilateral tonsillectomy and unilateral tongue base mucosectomy	Malignant	9	90	BMG, Monopolar Hook	No		Trial of 4 arms—poor access with 4th arm difficult to access tongue base with 4 arms.—abandoned 4th arm
23	Excision of tongue base lesion	Benign	10	25	BMG, Monopolar Hook	No		Trial of 4th arm. 4 instrument clashes when 4th arm in use
24	Excision of palatal lesion	Benign	9	8	BMG, Monopolar Hook	No		Trial of 4th arm. 4th BSU placed closer to bedside—improved superior access to superior palatal lesion
25	Excision of oropharyngeal lesion + unilateral tonsillectomy	Benign	8	34	BMG, Monopolar Hook	No		4th arm trialled successfully, no significant issues
26	Revision lateral oropharyngectomy	Malignant	9	120	BMG, Monopolar Hook	No		No issues encountered. Range of rotation of instrument beneficial
27	Unilateral tonsil biopsy	Malignant	8	25	BMG, Monopolar Hook	No		3 arm surgery. Performed by trainee. No issues
28	Bilateral tonsillectomy	Benign	7	33	BMG, Monopolar Hook	Secondary haemorrhage day 5 and day 7 post procedure. Managed conservatively	CD2	4 arm surgery right tonsillectomy 3 arm surgery left tonsillectomy 4th arm provides additional option for retraction but not of great benefit during tonsillectomy
29	Lateral oropharyngectomy	Malignant	14	115	BMG, Monopolar Hook	No		No issues
30	Unilateral tonsillectomy	Benign	6	23	BMG, Monopolar Hook	No		3 arm surgery. Performed by trainee. No issues

The median (interquartile range [IQR]) console time was 47.5 (33–105) minutes for all procedures. For benign surgery, the median console time was 40 (19–47.8) min. For malignant surgery, median console time was 104 (60–119.3) min. Figure 3 demonstrates console time sequentially by case.

The median setup time of the Versius system was 10 (7–13) min and a significant learning curve of setup time was experienced (Fig. 4).

### Evolution of operative approach

The study protocol was to complete a minimum of five benign cases prior to commencing cancer cases. During the initial cases optimisation of electrocautery settings and delivery required optimisation as this could not be evaluated preclinically. Initial electrocautery delivery was inconsistent, and it was found that a direct contact electrocautery grounding pad rather than a grounding gel mat was required for consistent delivery. Additionally, several power settings were trialled before an optimal setting was decided on. This process led the team to decide further benign cases were required before proceeding to cancer cases. 7 benign cases were performed prior to commencement of malignant procedures (Fig. 3. marker A).

### Instruments

Assessment of available instruments in conjunction with electrocautery settings occurred throughout (Table 3).

Due to lack of electrocautery and suboptimal size, the fenestrated grasper was reported to be suboptimal for use in TORS and was only trialled in 2 cases (1 and 6). The alternative grasping forceps (bipolar Maryland grasper, BMG) was found to be a more appropriate size for oropharyngeal use and was able to provide haemostasis however it was noted the instrument has limited grasping strength when retracting and the fine motor control required to maintain approximately 1 mm of jaw opening for effective bipolar delivery was challenging.

The Versius Monopolar hook and monopolar curved scissors were evaluated as primary cutting instruments in the absence of a monopolar spatula. Monopolar scissors were trialled in cases 1, 2, 3, 5, 9 and 10 and found to provide effective electrocautery, however, visualisation of the instrument tip was difficult due to the insulating sleeve of the instrument. The monopolar hook was more precise in delivery and the tip more easily visible. From case 11 onwards, the combination of monopolar hook and bipolar Maryland graspers became the standard operating instruments. Effective use of the monopolar hook was challenging in the tongue base. The curved tip requires the instrument to be bent at the wrist and occupies increasing the required working space.

### Bedside setup and introduction of 4 arm surgery

**Fig. 1:**
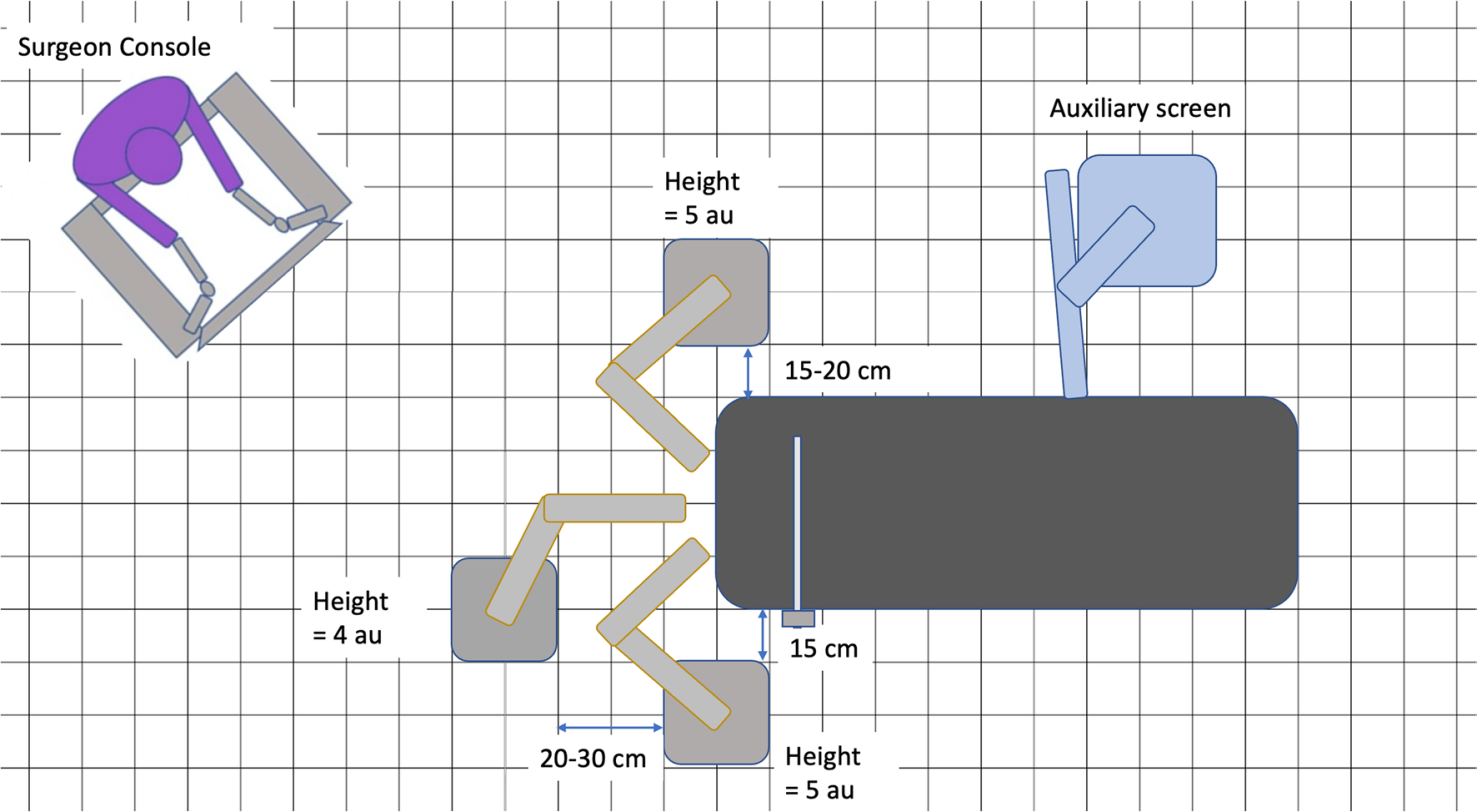
Schematic of Versius setup around the patient bedside showing surgeon positioned facing patient bedside to facilitate communication

**Fig. 2:**
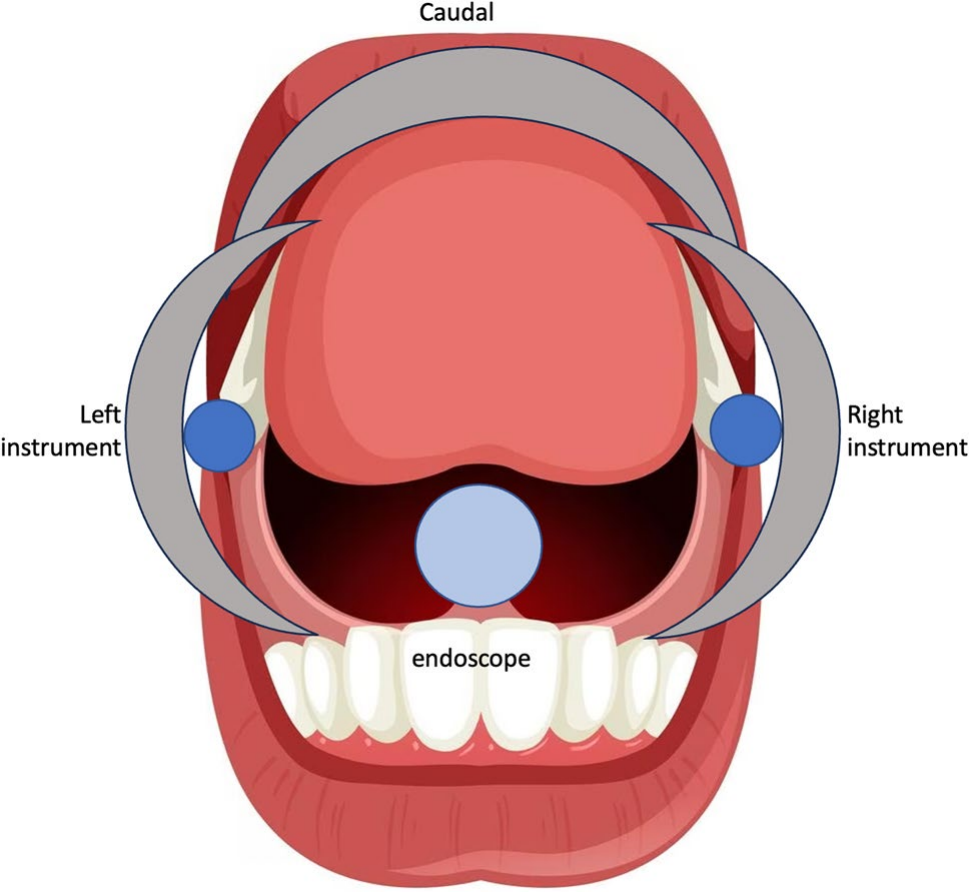
Schematic of oral cavity with check retractor in situ and placement of VPP for endoscope, left and right instruments

**Fig. 3:**
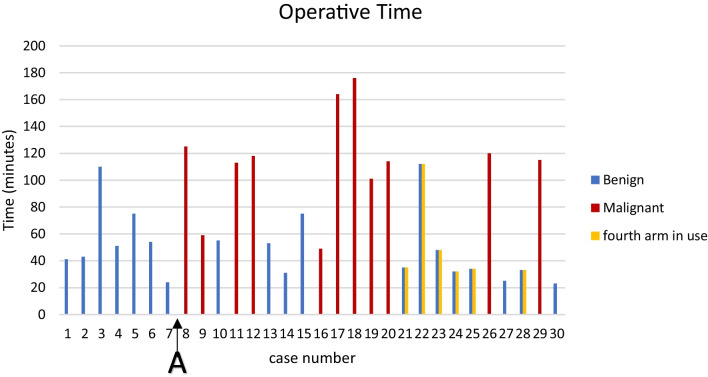
Operative times for benign, malignant and four-arm procedures

**Fig. 4:**
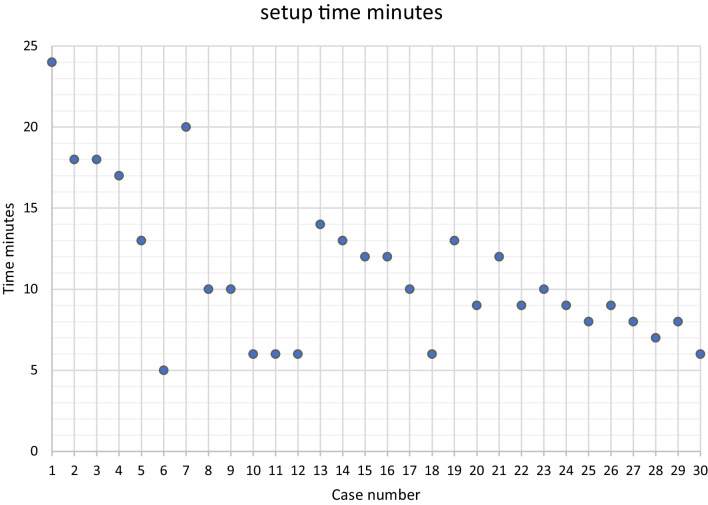
Versius setup time in minutes

Bedside setup of the surgical system remained consistent throughout the study and no significant changes were made to preclinical evaluation demonstrated in Figs. 1 and 2 until the introduction of a fourth bedside unit and a third surgical instrument after case 20 (Fig. 3. marker B). System setup time reduced greatly from 24 min to less than 10 min throughout the study period (Fig. 4).

**Fig. 5:**
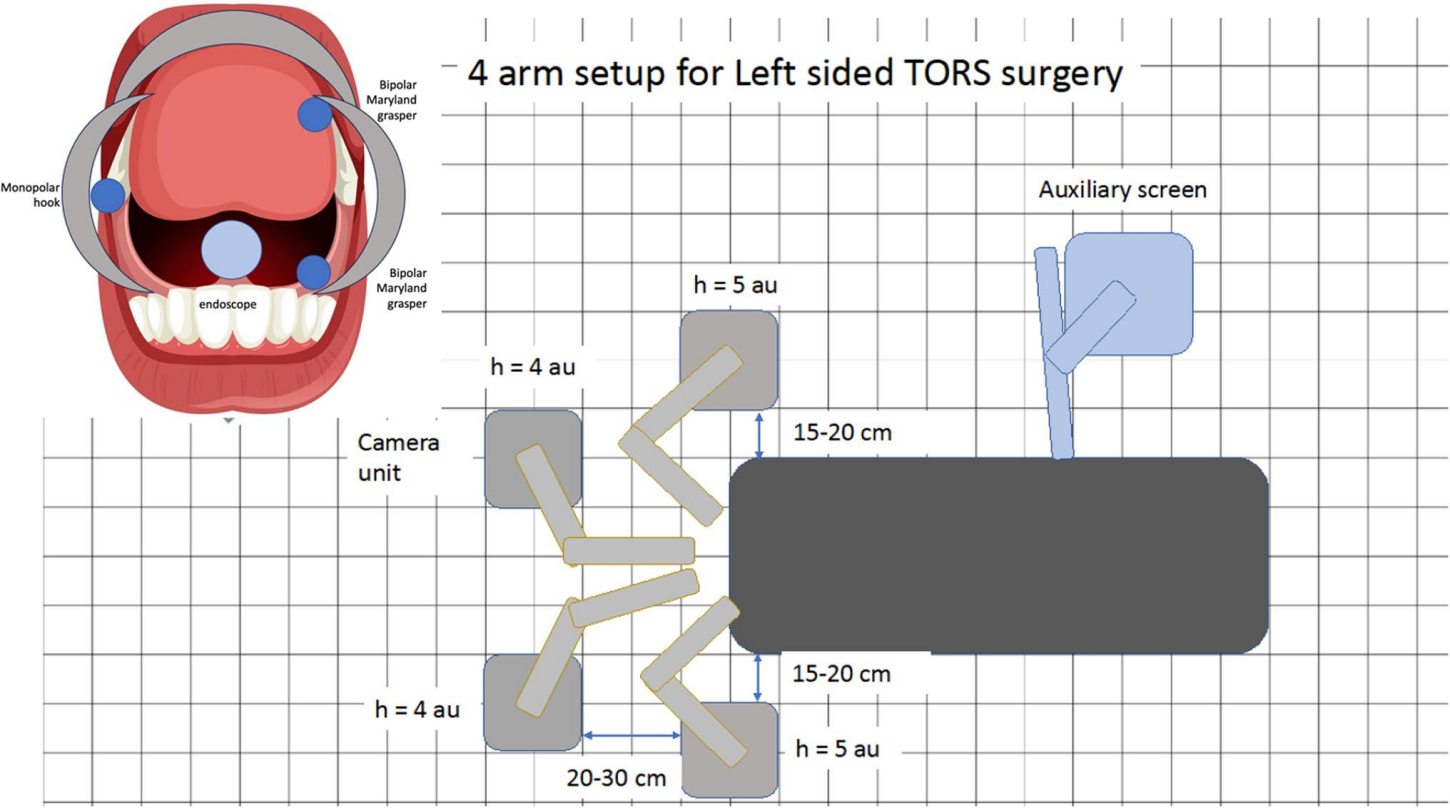
VPP placement and BSU placement for four-arm TORS with the Versius Surgical System

A fourth arm was introduced after case 20 to provide additional transoral retraction. In order to facilitate this the VPP placement of the instrument arms required amendment to minimise instrument clashes, maximise instrument mobility and maintain bedside surgeon access to the oral cavity. Figure 5 demonstrates room setup and VPP placement for fourth arm use. The use of a fourth arm was limited and found to increase instrument clashes when used in the tongue base (case 22) more than the proximal oropharynx.

## Discussion

This study describes the first in human clinical experience and case series of the Versius Surgical System (CMR Surgical, Cambridge, UK) for use in transoral robotic surgery. All 30 surgical procedures were successfully completed and the study demonstrates the feasibility of the system within the head and neck without significant complications. The 30 cases cover the significant majority of TORS procedures performed within the UK including tongue base mucosectomy for head and neck carcinoma for unknown primary, lateral oropharyngectomy for early oropharyngeal cancer and revision cancer surgery. 1 patient (case 3) experienced prolonged tongue numbness and dysgeusia following a 107-min procedure. This was the longest benign procedure of the cohort. During this procedure, there was a delay of 28 min due to electrocautery issues which contributed to a prolonged time under oral retraction. This may have contributed to the patients' post-procedure symptoms.

Unreliable electrocautery in the first five cases necessitated a delay in commencing cancer cases due to the increased risk of intraoperative bleeding and need for precise haemostasis. The study team performed extensive preclinical cadaveric assessment of the system however in this environment it is not possible to effectively evaluate electrocautery settings or the ability to effectively control bleeding. Preclinical live animal studies might have detected this issue.

Throughout preclinical evaluation the primary instruments used were the fenestrated graspers and monopolar hook and scissors [13]. When translated to the clinical environment it was apparent the lack of bipolar electrocautery was a significant limitation of the fenestrated graspers and necessitated the use of the BMG forceps. The monopolar hook was found to have significant advantages over the monopolar scissors as the electrocautery delivery was more precise and the tip easily visible. However, the curved nature of the monopolar hook exposes a large electrified area increasing the risk of inadvertent injury particularly at the heel of the hook. The Versius system instruments are afforded 360-degree rotational freedom whilst the surgeon is able to maintain comfortable wrist and hand positions [14]. This was found to be highly desirable and allowed superior access to challenging anatomical sites including the tongue base and deep aspects of the lateral oropharynx. The available Versius instruments were reported by the operating surgeon to be sub-optimal for TORS due to the large exposed area of the monopolar hook and limited grasping strength and precision of the bipolar Maryland forceps. This however does not appear to have impacted operative outcomes however is felt to have led to increased console time. The study team has reported to the manufacturer that the development of TORS-specific instrumentation including a monopolar spatula, optimised Maryland forceps and bipolar fenestrated graspers would enhance the surgical experience and aid with the adoption of brand agnostic robotic head and neck surgery.

Surgical console time was highly variable throughout the study and no clear surgical time learning curve was apparent. TORS is a heterogenous group of robotic assisted procedures especially within this cohort of benign, malignant and revision surgery and it is therefore more challenging to directly compare surgical times from one procedure to the next. Further work is required to establish the surgical learning curve for the Versius Surgical System in different transoral robotic surgery procedures. However, anecdotally from the participating surgical team, familiarity, confidence and understanding of the system improved greatly throughout the study. Setup times notably reduced and the team introduced fourth arm surgery and began to involve trainee surgeons as primary console operators.

4 arm robotic operating (camera and three instrument arms) within the oropharynx is currently not clinically possible with existing multiport robotic systems and has been limited to use with single port robotic systems such as the Da Vinci SP (Intuitive Inc. Sunnyvale, CA, USA). 4 arm operating has been reported to enhance surgical retraction, provide improved surgical exposure and reduce the workload of the bedside surgeon [15]. The Versius Surgical System has characteristics that allow four-arm operating that are not possessed by other multiport robotic systems. Each BSU can be placed individually around the bedside such to minimise proximal instrument and arm clashes and the BSU wrist size allows each arm to operate sufficiently close to one another to enable four-arm access to the oropharynx [13]. This study evaluated the use of four arms in six cases and found the additional retraction beneficial, however, to adequately utilise four surgical arms excellent transoral access was required and surgical access was more limited in distal sites including the tongue base. For four-arm surgery to be optimised with this system, instrument optimisation and miniaturisation would likely be required and further work is needed to define its scope and limitations.

The IDEAL recommendations for stages 1 and 2a were strictly followed in this study [16]. The sequential description of cases with an account of experience and iterative changes based on this provides a clear explanation of how our current approach evolved, benefiting other groups by allowing them to learn from our experience without having to recapitulate it. The planned progression from benign to malignant cases represents a prudent and ethically appropriate approach to innovation, with patient safety prioritised. The problems noted with instrumentation have been fed back to the manufacturer, and will lead to the production of modified instruments better suited to this type of surgery, whilst the early experience with 4 robotic arms has already allowed us to draw conclusions about the specific circumstances in which this may be beneficial. The surgical team feel that they now have a stable and clearly defined strategy for the use of Versius in TORS. This will allow further studies to proceed using a clearly defined, stable approach. IDEAL recommends multicentre studies in Stage 2b, involving a heterogeneous cohort of patients to allow evaluation of operator learning curves, establish how outcomes are affected by patient characteristics and technical variations, and promote consensus on the parameters for a definitive randomised trial. Such studies would require the formation of an interest group of TORS surgeons and the cooperation of the robot manufacturer.

## Conclusions

This IDEAL 1/2A prospective development study demonstrates that the Versius Surgical System (CMR Surgical, Cambridge, UK) is a viable robotic system for transoral robotic surgery and can be effectively utilised throughout the spectrum of TORS procedures. The system can provide sufficient transoral access to facilitate the use of 4 surgical arms. Optimisation of available robotic instruments is desirable to further enhance the transoral robotic surgery experience and aid wider dissemination of the system for TORS.

Further work in accordance with the IDEAL framework in the form of stage 2b exploratory studies and multicentre evaluation is required to further assess the suitability of Versius for wider adoption for transoral applications.

### Supplementary Information

Below is the link to the electronic supplementary material.

Supplementary file1 (PDF 241 KB)

Supplementary file2 (PDF 181 KB)
