# Supplementary material for: Prospective development study of the Versius Surgical System for use in transoral robotic surgery: an IDEAL stage 1/2a first in human and initial case series experience
Source: Eur Arch Otorhinolaryngol. 2024 Mar 26;281(5):2667–78. doi: 10.1007/s00405-024-08564-6 (PMC11023952; doi:10.1007/s00405-024-08564-6)
Supplement: Supplementary file 1 — Supplementary file1 (PDF 241 KB) [file 405_2024_8564_MOESM1_ESM.pdf]

Study Title: IDEAL STAGE 1/2A evaluation of transoral robotic surgery utilising the Versius Robotic System

Date and Version No: 11/08/2022: Version 2

**Chief Investigator:**	Mr Asit Arora, School of Biomedical Engineering and Imaging Sciences, King's College London and Guy's and St Thomas' NHS Foundation Trust
**Investigators:**	Jack Faulkner School of Biomedical Engineering and Imaging Sciences, King's College London and Guy's and St Thomas' NHS Foundation Trust
	Jean-Pierre Jeannon, Guy's and St Thomas' NHS Foundation Trust
	Professor Prokar Dasgupta King's College London and Guy's and St Thomas' NHS Foundation Trust
	Professor Sebastien Ourselin, School of Biomedical Engineering and Imaging Sciences, King's College London
**Sponsor:**	Guy's and St Thomas' NHS Foundation Trust

Chief Investigator Signature:

TABLE OF CONTENTS

1. KEY CONTACTS 4
2. ABBREVIATIONS 4
3. BRIEF SUMMARY 5
4. BACKGROUND AND RATIONALE 5
5. STUDY DESIGN 6
6. OUTCOME MEASURES 6
7. PARTICIPANT IDENTIFICATION 7
   7.1. Eligability Criteria 7
   7.2. Inclusion Criteria 7
   7.3. Exclusion Criteria 7
8. PROTOCOL PROCEDURES 7
   8.1. Recruitment 7
   8.2. Screening and Eligibility Assessment 7
   8.3. Informed Consent 8
   8.4. Blinding 8
   8.5. Study Intervention 8
   8.6. Baseline Assessments 9
   8.7. Subsequent Visits 9
   8.8. Early Discontinuation/Withdrawal of Participants 9
   8.9. Definition of End of Study 9
9. SAFETY REPORTING 10
   9.1. Definition of Serious Adverse Events 10
   9.2. Reporting Procedures for Serious Adverse Events 10
10. DATA MANAGEMENT 10
   10.1. Source Data 10
   10.2. Access to Data 10
   10.3. Data Recording and Record Keeping 11
11. ETHICAL AND REGULATORY CONSIDERATIONS 11
   11.1. Declaration of Helsinki 11
   11.2. Guidelines for Good Clinical Practice 11
   11.3. Reporting 11
   11.4. Participant Confidentiality 11

12. FINANCE AND INSURANCE 11
12.1. Funding 11
12.2. Insurance 12
13. PUBLICATION POLICY 12

1. KEY CONTACTS

**Chief Investigator**	Asit Arora
**Sponsor**	Guy's and St Thomas' NHS Foundation Trust<br>Guy's Hospital<br>Great Maze Pond<br>London<br>SE1 9RT
**Funder(s)**	CMR Surgical<br>194 Science park<br>Milton Road<br>Cambridge
**Clinical Trials Unit**	R&D Department<br>16th floor, Tower Wing<br>Guy's Hospital<br>Great Maze Pond<br>London<br>SE1 9RT

2. ABBREVIATIONS

CI	Chief Investigator
GCP	Good Clinical Practice
GP	General Practitioner
ICF	Informed Consent Form
NHS	National Health Service
PI	Principal Investigator
PIL	Participant/ Patient Information Leaflet
R&D	NHS Trust R&D Department

SOP	Standard Operating Procedure
TORS	Transoral Robotic Surgery
MDADI	MD Anderson Dysphagia Inventory

3. BRIEF SUMMARY

This is a study evaluating the use of the Versius Robotic System to perform Transoral Robotic Surgery in a clinical setting. The primary outcome is the completion of transoral robotic procedures. Secondary outcomes are operative times, perioperative complications, margin status for cancer cases and 3 month post-operative functional outcomes. Any modifications to technique, indication or equipment which appear necessary or advisable in the light of experience during the study will be documented and explained in alignment with IDEAL Recommendations for Stage 2a [REF]

Commented [PM1]: And learning? I have added some quick suggestions

4. BACKGROUND AND RATIONALE

Transoral robotic surgery was first established in 2005 and has become a mainstay in the treatment paradigm of head and neck pathology. Since its inception a single family of robotic platforms (Da Vinci, Intuitive Inc.) has been the predominant robotic system utilised in TORS. However new robotic systems are entering the market.

The Versius Surgical System from CMR Surgical, Cambridge, UK is a commercially available robotic system which is CE marked for use in other surgical specialities and is in clinical use. The system utilises robotic arms mounted on individual bedside units (BSU's) which can be independently configured around the bedside. The surgeon operates via an open console, wearing specialised glasses for 3d vision. (Thomas et al., 2021) (Kelkar et al., 2020)

Pre-clinical cadaveric evaluations have been conducted with this platform in accordance with the IDEAL-D framework stage 0. These suggest TORS is feasible to be performed with Versius and first in human studies are appropriate. (Faulkner et al., 2021)

Commented [PM2]: More detail? How many: any additional preparatory work which should be mentioned?

IDEAL provides a framework for reporting surgical innovation. Stage 1 is an initial report, with 2a focusing on technical details, learning and feasibility from a small single-centre experience. Initial clinical experience with new operations or new technology frequently provides important opportunities for learning and improvement. IDEAL recommends that these should be reported fully and transparently in accordance with basic ethical principles.

This study proposes a first in human evaluation of the Versius Surgical System in accordance with IDEAL stage 1 and 2a principles.

5. STUDY DESIGN

This study constitutes an IDEAL stage 1/2a evaluation using a single centre prospective case series on the use of a new robotic surgical system for performing transoral robotic surgery. It aims to recruit 15 patients. To maximise safety initial recruitment will target patients with benign pathology before proceeding to cancer cases where appropriate. This study will take place at a UK tertiary head and neck centre with an extensive current robotic practice.

Study Type	Sequential case series IDEAL stage 1/2a
Estimated Enrolment	15 participants
Allocation	N/A
Interventional Model	Single Group Assignment
Masking	None (open label)
Primary Purpose	Treatment
Official Title	IDEAL STAGE 1/2A evaluation of transoral robotic surgery utilising the Versius Robotic System
Estimated study start date	01/11/2022
Estimated primary completion date	01/01/2023
Estimated study completion date	01/02/2023

Arms and interventions

Arm	Intervention
Experimental:<br>Prospective single arm study to investigate the use of a new robotic surgical system on efficacy, safety and functional performance during transoral robotic surgery	Oropharyngeal resection will be performed in accordance with recognised robotic technique utilising the Versius Surgical System.<br>The use of an additional trial endoscope stabilisation device may be utilised if indicated by operating surgeon.

6. OUTCOME MEASURES

Primary Outcome Measure

1. Successful completion of Transoral Robotic Surgery procedures performed using the Versius Surgical System.
   Successful completion is defined by operating surgeon.

Secondary Outcome Measures

Commented [PM3]: And learning

1. Rate of perioperative complications [time frame 30 days]
   All intraoperative and postoperative complications occurring within 30 days after discharge will be graded according to the Clavien-Dindo classification.

2. Intraoperative time
   Robotic docking time and operative times will be collected
3. Margin status for cancer cases
   Surgical margins for cancer resections will be determined and reported as per routine procedure by the centres pathology department.
4. Functional outcomes
   Preoperative and 30 day post operative MD Anderson Dysphagia Inventory (MDADI) will be conducted to report functional swallow outcomes.
5. Documentation of learning
   Any experience which suggests that changes to the technology, its use in practice, patient selection criteria or surgical technique are necessary or desirable will be fully explained together with an account of any modifications or adaptations performed in response.

7. PARTICIPANT IDENTIFICATION

7.1. Eligibility Criteria

Patients with benign and early malignant oropharyngeal pathology who would as part of their regular treatment be ordinarily offered TORS will be considered for recruitment into the study. The first 5 cases will be reserved for less complex cases as defined by the clinical team. If the appropriate due to successful completion and absence of adverse events more complex cases may be considered.

7.2. Inclusion Criteria

1. Participant is willing and able to give informed consent for participation in the study
2. Male or Female
3. Aged 18 years or above.
4. Patient suitable for TORS as part of their routine clinical care.

7.3. Exclusion Criteria

1. Vulnerable population including prisoners.
2. Severe concomitant illness that drastically reduces life expectancy or increases risk of therapeutic intervention

8. PROTOCOL PROCEDURES

8.1. Recruitment

Participant recruitment will occur during routine outpatient appointments conducted by the practicing clinical team at the surgical centre where the study is taking place.

8.2. Screening and Eligibility Assessment

All patients will be screened by clinical staff to ensure fulfilment of eligibility criteria without exception. Patient screening will occur prior to consent.

8.3. Informed Consent

Commented [PM4]: Explanation of state of the art and unknown unknowns

Written and verbal versions of the Participant Information and Informed Consent will be presented to the participants detailing no less than: the exact nature of the study; what it will involve for the participant; the accumulated experience with the technique and equipment to date, the implications and constraints of the protocol; the known side effects and any risks involved in taking part, including the possible existence of unknown risks. It will be clearly stated that the participant is free to withdraw from the study at any time for any reason without prejudice to future care, without affecting their legal rights, and with no obligation to give the reason for withdrawal.

The participant will be allowed as much time as wished to consider the information, and the opportunity to question the Investigator, their GP or other independent parties to decide whether they will participate in the study. Written Informed Consent will then be obtained by means of participant dated signature and dated signature of the person who presented and obtained the Informed Consent. The person who obtained the consent must be suitably qualified and experienced and have been authorised to do so by the Chief/Principal Investigator. A copy of the signed Informed Consent will be given to the participant. The original signed form will be retained at the study site.

8.4. Blinding

This is an unblinded “open label” study and no blinding will occur.

8.5. Study Intervention

Study devices

The Versius Surgical System (CMR Surgical) is a software-controlled, electromechanical system designed to be utilised by surgeons to perform minimally invasive surgery. Versius consists of a surgeon console, a vision cart, individual bedside units that control surgical instruments and a 3d endoscope and accessories.

An additional accessory device that holds the robotic endoscope has been developed by the trial team as outlined by pre-clinical work. The device is non-patient contacting.

System training

All participating surgeons and all members of the surgical team will undertake the required clinical training to utilise Versius as defined by the device manufacturer CMR Surgical. This includes but is not limited to completion of online training modules, simulation training and a 2.5 day onsite team training.

Intervention

The Versius system will be positioned and utilised as per optimal pre-clinical findings and all procedures will be performed in accordance with recognised surgical methods. If study findings suggest a more

optimal system setup then appropriate adjustments will be made and reported, as per IDEAL Recommendations. If deemed by the surgical team that the additional trial endoscopic holder is required, then the use of this will also be assessed.

Commented [PM5]: All modificaitons to be explained and results commented on as per 2a

8.6. Baseline Assessments

All patients enrolled in the study will undergo pre-procedure functional assessment via the MD Anderson Dysphagia Inventory performed through a patient completed questionnaire. Patient demographics and biometrics will be recorded.

8.7. Subsequent Visits

Patients will undergo a 30 day post procedure review to ensure no adverse events have been unreported and repeat functional assessment. Patients consent to continue to participate in the study will be confirmed at each visit.

8.8. Early Discontinuation/Withdrawal of Participants

During the course of the study a participant may choose to withdraw early from the study treatment at any time. This may happen for several reasons, including but not limited to:

- The occurrence of what the participant perceives as an intolerable AE.
- Inability to comply with study procedures
- Participant decision

Participants may choose to stop treatment and/or study assessments but may remain on study follow-up.

Participants may also withdraw their consent, meaning that they wish to withdraw from the study completely.

In addition, the Investigator may discontinue a participant from the study treatment at any time if the Investigator considers it necessary for any reason including, but not limited to:

- Ineligibility (either arising during the study or retrospectively having been overlooked at screening)
- Significant protocol deviation
- Clinical decision

The withdrawal and reason for withdrawal will be recorded in the CRF.

8.9. Definition of End of Study

The end of study period is defined by the 30 day follow-up of the last enrolled patient.

9. SAFETY REPORTING

All serious adverse events (SAE) that occur during the trial period from the start of intervention to completion of 30 day follow-up will be reported to the oversight committee- the surgical oncology directorate committee and the new intervention procedures committee as soon as practically possible.

On the occurrence of any SAE further enrolment and participation of the study will be temporarily or permanently suspended until investigated. In the event of a SAE the participant will receive ongoing follow-up until the resolution or stabilisation of the event.

9.1. Definition of Serious Adverse Events

A serious adverse event is any untoward medical occurrence that:

- results in death
- is life-threatening
- requires inpatient hospitalisation or prolongation of existing hospitalisation
- results in persistent or significant disability/incapacity
- consists of a congenital anomaly or birth defect.

Other ‘important medical events’ may also be considered a serious adverse event when, based upon appropriate medical judgement, the event may jeopardise the participant and may require medical or surgical intervention to prevent one of the outcomes listed above.

NOTE: The term "life-threatening" in the definition of "serious" refers to an event in which the participant was at risk of death at the time of the event; it does not refer to an event which hypothetically might have caused death if it were more severe.

9.2. Reporting Procedures for Serious Adverse Events

A serious adverse event (SAE) occurring to a participant should be reported to the REC that gave a favourable opinion of the study where in the opinion of the Chief Investigator the event was ‘related’ (resulted from administration of any of the research procedures) and ‘unexpected’ in relation to those procedures. Reports of related and unexpected SAEs should be submitted within 15 working days of the Chief Investigator becoming aware of the event.

10. DATA MANAGEMENT

10.1. Source Data

Source data will be collected from clinical record forms, medical records and completed patient questionnaires for MDADI.

10.2. Access to Data

Direct access will be granted to authorised representatives from the Sponsor and host institution for monitoring and/or audit of the study to ensure compliance with regulations.

10.3. Data Recording and Record Keeping

All trial data will be entered on paper CRFs and stored securely at the participating trial site.

The participants will be identified by a unique trial specific number and/or code in any database. The name and any other identifying detail will NOT be included in any trial data electronic file.

11. ETHICAL AND REGULATORY CONSIDERATIONS

11.1. Declaration of Helsinki

The Investigator will ensure that this study is conducted in accordance with the principles of the Declaration of Helsinki.

11.2. Guidelines for Good Clinical Practice

The Investigator will ensure that this study is conducted in accordance with relevant regulations and with Good Clinical Practice.

11.3. Oversight committee

The investigator will seek and ensure approval for the study from the clinical oversight committees, - Surgical oncology directorate committee and the robotic steering committee prior to commencement of the study.

11.4. Reporting

The CI shall submit an End of Study notification and final report to the Oversight committees, sponsor and funder.

11.5. Participant Confidentiality

The study will comply with the General Data Protection Regulation (GDPR) and Data Protection Act 2018, which require data to be de-identified as soon as it is practical to do so. The processing of the personal data of participants will be minimised by making use of a unique participant study number only on all study documents and any electronic database(s). All documents will be stored securely and only accessible by study staff and authorised personnel. The study staff will safeguard the privacy of participants’ personal data.

12. FINANCE AND INSURANCE

12.1. Funding

Funding for surgical team training and support has been provided by CMR Surgical.

12.2. Insurance

NHS bodies are legally liable for the negligent acts and omissions of their employees. If you are harmed whilst taking part in a clinical research study as a result of negligence on the part of a member of the study team this liability cover would apply.

Non-negligent harm is not covered by the NHS indemnity scheme.

13. PUBLICATION POLICY

The Investigators will be involved in reviewing drafts of the manuscripts, abstracts, press releases and any other publications arising from the study. Authors will acknowledge that the study was supported by CMR Surgical. Authorship will be determined in accordance with the ICMJE guidelines and other contributors will be acknowledged.

References

Faulkner, J., Arora, A., Swords, C., Cook, E., Rajangam, A., & Jeannon, J. (2021). Pre-clinical evaluation of a novel robotic system for transoral robotic surgery. *Clinical Otolaryngology: Official Journal of ENT-UK; Official Journal of Netherlands Society for Oto-Rhino-Laryngology & Cervico-Facial Surgery,*

Kelkar, D., Borse, M., Godbole, G., Kurlekar, U., Dinneen, E., Stevens, L., & Slack, M. (2020). 43: First-in-human clinical trial of a new robot-assisted surgical system for total laparoscopic hysterectomy. *American Journal of Obstetrics & Gynecology, 222*(3), S800-S801.

Thomas, B. C., Slack, M., Hussain, M., Barber, N., Pradhan, A., Dinneen, E., & Stewart, G. D. (2021). Preclinical evaluation of the Versius Surgical System, a new robot-assisted surgical device for use in minimal access renal and prostate surgery. *European Urology Focus, 7*(2), 444-452.
