# Supplementary material for: Prospective development study of the Versius Surgical System for use in transoral robotic surgery: an IDEAL stage 1/2a first in human and initial case series experience
Source: Eur Arch Otorhinolaryngol. 2024 Mar 26;281(5):2667–78. doi: 10.1007/s00405-024-08564-6 (PMC11023952; doi:10.1007/s00405-024-08564-6)
Supplement: Supplementary file 2 — Supplementary file2 (PDF 181 KB) [file 405_2024_8564_MOESM2_ESM.pdf]

Guy's and St Thomas' NHS Foundation Trust

Patient information document:

IDEAL stage I/IIa evaluation of the Versius Surgical System for trans-oral robotic surgery.

Sponsor: Guy's and St Thomas' NHS Foundation Trust

Guy's Hospital

Great Maze Pond

London

SE1 9RT

Principle investigator: Mr Asit Arora

Study team contact details

Mr Asit Arora – Consultant ENT Head &Neck surgeon	020 71882213
Nicola Parker – Head and Neck Service Manager	020 71887691

Introduction

You are being invited to take part in the evaluation of the Versius Surgical System for use in transoral robotic surgery. Your participation is completely voluntary. You can choose not to take part and you can withdraw from the study at any time.
Before you make your decision to take part, you will need to know about the study. Please take time to read and understand this information leaflet. Your surgical team will also discuss this study in detail with you. Please ask them any questions or raise any concerns that you may have with them.
You should decide to take part in this study only when:

- Your surgical team have explained the details of the study to you
- You have understood why we are doing this study
- You have understood what you will have to do while taking part in this study
- You have understood the risks and benefits of the study
- You have understood what other treatments options you may have

You may want to talk with your family, friends and/or your family doctor to help make your decision.
If you decide to take part in the study, you (or your legally acceptable representative) will be asked to sigh the Informed Consent Form. You will also receive a copy of this document for your records.

What is the Versius Surgical Robotic System?

The Versius Surgical Robotic System (we will call it Versius for the rest of this booklet) is a new surgical robot. It is controlled by a surgeon throughout the operation. A camera is attached to one of the robotic arms while the other arms have surgical instruments attached to them. The surgeon uses hand controllers (joysticks) to control the arms and can perform surgery with them. Versius has been developed and built by CMR Surgical Limited, a UK based and registered company.

What is the purpose of this study?

This study is looking to see if Versius can be used to perform transoral robotic surgery. Transoral robotic surgery or TORS is a more sophisticated way of performing surgery that can make it easier to recover from and benefit patients in terms of better functional outcome such as swallowing after surgery. We have been performing transoral robotic surgery at Guy's for over 4 years using a different a robotic system (Da Vinci from Intuitive Incorporated a US based company).

Versius is another surgical robot that we already have here at Guy's. It is currently used to perform urological surgery such on the prostate, gynaecology surgery such as on the womb and colorectal surgery on the bowel and is in use in several other centres in the UK and rest of the world. We hope that it can also be used to help perform transoral surgery.

The robot does not carry out any procedure on its own. A surgeon sitting at a console near the patient controls it. The surgeon is then able to 'drive' robotic arms, which are connected to 'keyhole' instruments within the patient's mouth.

The advantage of robotic assisted surgery is that it allows the surgeon to perform complex manoeuvres, such as completely removing tumours without disturbing the tumour itself. This is something that is normally very difficult to do using standard transoral instruments.

Our team have conducted multiple "pre-clinical" evaluations of Versius with many expert robotic surgeons. The conclusion of these studies is that we are ready to proceed to first in human evaluation. Our hope is that this will lead to wider availability of trans oral surgery for more patients.

How have the study team been trained to use the Versius Surgical Robotic System

All the study doctors and surgical team have been trained to use Versius by qualified clinical training teams from CMR Surgical Limited. All surgical staff involved must have completed the comprehensive training programme and have practiced using the robot safely and professionally before taking part in this study.

The team consists of two lead surgeons, Mr Asit Arora and Mr Jean-Pierre Jeannon. Both Mr Arora and Mr Jeannon have a lot of experience in TORS. Mr Arora was part of the team that pioneered the technique in the UK and this was the focus of his PhD thesis. He established the robotic programme here at Guy's and is the robotic lead in head and neck surgery at Guy's. Mr Jean-Pierre Jeannon is also a very experienced Head & Neck cancer surgeon with extensive robotic experience. Both surgeons have been closely involved in the pre-clinical testing of Versius.

Why am I being asked to participate?

You are being invited to take part in this study because your doctor has recommended you for transoral robotic surgery and you are eligible to have surgery using Versius. Other patients like you will also be invited to take part.

What will happen to me if I decide to take part?

If you decide to take part in the study, you will sign the Informed Consent Form to say you are willing to participate and you understand what is involved.
You can change your mind and withdraw at any point.
Participation in this study will not change the treatment options you are offered as part of your care.
If you decide not to participate or to withdraw, you will be offered transoral robotic surgery with another robot if suitable or you will be offered non-robotic treatment options.

Study visits

If you agree to take part, you will undergo an initial assessment to confirm your eligibility and consent to take part. During this the surgeon will explain the surgical procedure and the risks involved. This will occur before the day of your operation. You will also be asked to complete a questionnaire regarding your swallowing ability as this is an important marker after these operations.

On the day of the surgery the surgeon will again explain the surgical procedure to you and any associated risks and document this discussion and confirm your consent to participate. During your hospital stay you will be seen by the surgical team and your health status and recovery progress noted.
30 days after surgery you will attend an appointment at the clinic in the hospital. You will be asked a few questions regarding your health and recovery. You will receive a clinical examination to assess your healing of the surgical site after your discharge from hospital. You will also be asked to complete another swallowing questionnaire.

The surgical procedure

Transoral robotic surgery is a group of operations to where a robotic system is used to remove tissue such as tumours from a patients throat in areas such as the tonsils or back of the tongue. It is performed by placing robotic instruments through the patients' mouth, avoiding cuts on the skin and improving recovery compared to open procedures. The advantage of robotic assisted surgery is that it allows the surgeon to perform complex manoeuvres, such as completely removing tumours without disturbing the tumour itself. This is something that is normally very difficult to do using standard transoral instruments. It was first introduced in 2005 and is a well-recognised group of procedures.
The surgical team will speak to you about the specific operation that you will be undergoing and answer any questions you have on this.

Your surgery with Versius

Versius is a new "next generation" robotic system that has differences to current surgical robots but it will be used to perform the same recognised procedures. It simply offers a new tool with which to perform the surgery. You will receive a general anaesthetic which means you will be fully asleep for the duration of your surgery. The surgeon will sit at the 'console' and control the robot through hand controllers to perform the surgery. Any tissue removed will be removed through your mouth without making cuts in the skin. Depending on the complexity of your procedure it can take between 1 and 3 hours to complete. Some patients are able to go home the same day after surgery but those that have more complex surgery will be required to stay for at least 1 night.
Most patients are able to start eating and drinking a few hours after surgery.

What are the potential risks of taking part in this study?

All complications that may happen during transoral robotic surgery with other robotic systems are possible with the Versius system. Your surgeon will talk to you about the risks specific to your procedure more thoroughly. Some of the risks generally involved with TORS surgery and are outline below in the "Risks of transoral robotic surgery" section. There could also be some risks that associated with performing robotic surgery with the Versius system as detailed below:

1. Versius is a new system and even the most experienced surgeons need to learn how to use it safely in patients. Your surgery will be performed by a surgeon who has completed the official Versius training and practiced the surgery before with Versius.

The study team has been performing transoral robotic surgery for over 4 years and has been involved in assessing the Versius robot of clinical use. Additionally, your surgical team has completed a training programme specific to Versius to maximise their knowledge and experience of the system.

2. Unexpected risks - Versius is a new robotic system that has not been used for TORS in patients before. Due to this there may be risks associated that are not yet known. Whilst the surgical team have conducted multiple assessments of the system and believe it can be safely used in TORS there may be some unpredictable and unknown risks.
3. In the event that there is a problem with the system during your surgery that can not be resolved, such as malfunction with the system the surgeon will put your care and safety first and may choose to remove Versius complete your procedure by other means. This may involve using a different robotic system or other techniques. In this event there may prolong your surgery and increase your risk of complications.

Risks of transoral robotic surgery (All robotic systems)

As with all surgery there are risks associated with your procedure regardless of what robotic system is used.
Your surgeon will explain the specific risks to your operation in person and in detail before you agree to take part. Below is a summary of the most common risks associated with transoral robotic surgery. Along with anaesthetic risks a list of possible complications include but are not limited to:
Pain – pain after TORS is very common regardless of technique. You will be discharged home with prescribed pain relief that you should take as per the doctors recommendation.
Infection – this can occur after any operation in your throat. You will be discharged on a course of antibiotics to reduce this risk.
Bleeding – bleeding can occur shortly after surgery or can be delayed by up to 2 weeks. If you experience any bleeding you are advised to attend your nearest accident and emergency department. Minor bleeding can occur before a heavier bleed that may require treatment in the operating theatre.
Damage to other structures – other structures within the mouth can occasionally be injured during surgery. These including teeth, lips, tongue, the jaw and other nearby structures.

Other potential complications you should be aware of:
Change in taste – in some cases following surgery patients may experience altered taste, this may be temporary and rarely permanent.
Nasal regurgitation – it is possible to after throat surgery that patients experience regurgitation of food and drink into the back of their nose on swallowing.
Swallowing difficulty – removal of tissue from your throat can sometimes cause difficulty swallowing. This may be temporary or permanent.
Need for further procedures – Sometimes if tumours are incompletely or closely removed some patients will be recommended to undergo further surgery to remove more tissue.
Risk of permanent injury or death – permanent injury from surgery is rare but do occur. Death is extremely rare from TORS surgery. Pre-existing medical conditions may increase your risk of complications. A full medical history will be taken from you and careful examinations will be carried out by your doctor prior to your surgery to minimise any risks.

Are there any alternatives to TORS?

Yes. Your doctor will talk to you about other surgery or treatment options that may be available to you instead of taking part in this study. The alternative to TORS with Versius is TORS with another robotic system that has been in use for many years. If you do not wish to have robotic surgery, there may be conventional surgery available or other options for you. Your surgeon will explain these to you. You do not need to take part in this study and can decide which treatment option you prefer after discussion with your doctor.

What are the benefits of TORS?

There are many benefits of robotic transoral surgery which include but are not limited to:
Improved surgical precision
Improved functional outcomes such as swallowing ability compared to open surgery
Earlier discharge from hospital compared to open surgery
Better clinical outcomes

However, we do not know if by taking part in this study and having an operation with Versius you will benefit from all or part of those. By taking part in the study you will help future patients by adding to what is known about Versius and if it can be used in TORS.

What if something goes wrong

Your surgical team will make every effort to prevent any harm to you during this study. If you feel you have been injured during this study you must contact your study team immediately so that they can ensure you receive the necessary care. In the event of any harm to a patient, this will be fully investigated in accordance with the trust's adverse events procedures. In the event of an adverse event you will be put in contact with the Patient Advice and Liaison service (PALS) who will assist you further.

What if new information becomes available about the study?

During the study we may find more information that could be important to you. The study doctor or team will tell you about any new important findings that relate to you taking part. This study aims to build and learn as it progresses. The Study team will be open, honest and transparent about the study findings as it progresses and will inform you of any issues that may have occurred with earlier participants.

Will all study participants be treated the same?

This study is part of what is called and IDEAL stage IIa study. What this means is that as the study develops the study team learns from the results and can adjust its procedure to maximise benefit to patients. You will be informed of any changes that have been made throughout the study and how this may impact your care.

What will happen If I don't want to carry on with the study?

Taking part in this study is entirely up to you. If you decide to take part but change your mind, you are free to leave the study at any time. Leaving the study will not affect the care you receive.

What information will be collected?

The study team will collect data on your surgical procedure and your recovery. This will involve video and photo documentation as well as written records.

Will my information be kept private and secure?

Yes. The study team have taken all reasonable measures to keen your data confidential, private and protected.
Information collected for the study will be given a unique identifier (ID number). Only the study team will know what ID number refers to you.
Any publication or report resulting from this study will not contain any identifiable or personal information.

Where will my data be stored?

Your information will be recorded and securely stored. A secure electronic database will record your reports and data. Additionally, a data capture system built into the Versius robot will record and securely store information about your surgery. Access to the data will be limited to members of the study team. Access may be given to regulatory authorities for auditing or inspection on request.
Any paper records will be securely stored within your patient notes. All personal data will be collected and stored in accordance with the Data Protection Act 2018.

Who has reviewed and approved the study?

This study has been registered and approved by the Guy's and St Thomas' NHS Foundation Trust Surgical Oncology Directorate and the Robotic Surgery Project Board. These boards will provide oversight of the study for its duration.

What if I have questions about the research study and my rights?

You should feel free to ask any questions about this study at any time. Your study doctor will try and fully answer any questions that you may have before or during the study.

Photographs/Recordings

Still photographs and video recordings of your surgery will be taken for documentation. These may be used in subsequent publications or for educational purposes. Every effort will be made to ensure you can not be identified in these.

Informed consent form

IDEAL stage I/IIA evaluation of the Versius Surgical System in Transoral Robotic Surgery

Patient Name: ……………………………………………

Patient Initials: ……………………………………………

Date of Birth: ……………………………………………

Patient Address: ………………………………………………………………………………………

………

By signing this form, you (or your legally acceptable representative) agree to take part in the study and agree to the below statements:

I, ______________________________ believe that the surgical procedure listed above is the best option for me. My situation is not an emergency and I understand that I have other options that include not having surgery. I understand that complications, bad outcomes and unexpected events may occur during surgery and recovery. I understand that at no point have I been guaranteed any outcomes or results. I have been given the opportunity to ask all my questions and I believe that I have all the information necessary to make a reasonable decision. Therefore, I elect to give my consent and proceed with the above surgery as witnessed by my signature below.

1. I confirm I have read and understood the information sheet for the above study and have had the opportunity to ask questions.
2. I understand my participation in the study is voluntary and that I am free to withdraw at any tim, without giving any reason, without my medical care or legal rights being affected.
3. I understand and commit to attending study visits both before and after my surgery as directed by the study and surgical team.
4. I Understand that personal information about me will be stored on the study database.
5. I agree that photos and videos from my surgery can be collected and used for presentation, publication and education following participation in this study.
6. I understand that data from this study may be published and shared with third parties and I understand that the risk of me being identified as a participant is negligible.
7. I understand that regulatory authorities and study staff may have direct access to my medical records.
8. I understand that I will not receive any money or compensation or benefits from the use of information acquired and developed through taking part in this study.
9. I agree to take part in this study.

10. I understand that my information will be held securely in accordance with the Data protection act 2018 and consent to share my information with the parties involved in this study.

Name of Patient ……………………………………………

Signature of Patient ……………………………………………

Date ……………………………………………

Name of legally acceptable representative (if required) ……………………………………………

Signature of legally acceptable representative ……………………………………………

Date ……………………………………………

Name of Investigator ……………………………………………

Signature of Investigator ……………………………………………

Date ……………………………………………

(Copy of the patient information sheet and Informed Consent Form will be provided for patients own records)

**Mr Asit Arora – Consultant ENT Head &Neck surgeon**	**020 71882213**
**Nicola Parker – Head and Neck Service Manager**	**020 71887691**

Pharmacy Medicines Helpline If you have any questions or concerns about your medicines, please speak to the staff caring for you or call our helpline. **t:** 020 7188 8748 9am to 5pm, Monday to Friday

Patient Advice and Liaison Service (PALS) To make comments or raise concerns about the Trust's services, please contact PALS. Ask a member of staff to direct you to the PALS office or: **t:** 020 7188 8801 at St Thomas' **t:** 020 7188 8803 at Guy's **e:** pals@gstt.nhs.uk

Language Support Services If you need an interpreter or information about your care in a different language or format, please get in touch using the following contact details. **t:** 020 7188 8815 **fax:** 020 7188 5953

NHS 111 Offers medical help and advice from fully trained advisers supported by experienced nurses and paramedics. Available over the phone 24 hours a day. **t:** 111

NHS Choices Provides online information and guidance on all aspects of health and healthcare, to help you make choices about your health. **w:** www.nhs.uk

Become a member of your local hospitals, and help shape our future
Membership is free and it is completely up to you how much you get involved. To become a member of our Foundation Trust, you need to be 18 years of age or over, live in Lambeth, Southwark, Lewisham, Wandsworth or Westminster or have been a patient at either hospital in the last five years. To join: **t:** 0848 143 4017 **e:** members@gstt.nhs.uk **w:** www.guysandstthomas.nhs.uk
